# Improvement of Catalytic Activity and Thermostability of Alginate Lyase VxAly7B-CM via Rational Computational Design Strategies

**DOI:** 10.3390/md23050198

**Published:** 2025-05-01

**Authors:** Xin Ma, Ke Zhu, Kaiyang Wang, Wenhui Liao, Xiaohan Yang, Wengong Yu, Weishan Wang, Feng Han

**Affiliations:** 1 School of Medicine and Pharmacy, Ocean University of China, Qingdao 266003, China; xma1221@163.com (X.M.);; 2 Laboratory for Marine Drugs and Bioproducts, Qingdao Marine Science and Technology Center, Qingdao 266237, China; 3 Key Laboratory of Marine Drugs, Ministry of Education, Qingdao 266003, China; 4 Shandong Key Laboratory of Glycoscience and Glycotherapeutics, Qingdao 266003, China; 5 State Key Laboratory of Microbial Diversity and Innovative Utilization, Institute of Microbiology, Chinese Academy of Sciences, Beijing 100101, China; 6 University of Chinese Academy of Sciences, Beijing 100049, China; 7 Beijing Key Laboratory of Genetic Element Biosourcing & Intelligent Design for Biomanufacturing, Beijing 100101, China

**Keywords:** alginate lyase, alginate oligosaccharides, thermostability, rational design, molecular dynamics simulation

## Abstract

Alginate lyase degrades alginate through the β-elimination mechanism to produce alginate oligosaccharides (AOS) with notable biochemical properties and diverse biological activities. However, its poor thermostability limits large-scale industrial production. In this study, we employed a rational computational design strategy combining computer-aided evolutionary coupling analysis and ΔΔG_fold_ evaluation to enhance both the thermostability and catalytic activity of the alginate lyase VxAly7B-CM. Among ten single-point mutants, the E188N and S204G mutants exhibited increases in *T*_m_ from 47.0 °C to 48.9 °C and 50.2 °C, respectively, with specific activities of 3701.02 U/mg and 2812.01 U/mg at 45 °C. Notably, the combinatorial mutant E188N/S204G demonstrated a Δ*T*_m_ of 5 °C and an optimal reaction temperature up to 50 °C, where its specific activity reached 3823.80 U/mg—a 31% increase. Moreover, its half-life at 50 °C was 38.4 h, which is 7.0 times that of the wild-type enzyme. Protein structural analysis and molecular dynamics simulations suggested that the enhanced catalytic performance and thermostability of the E188N/S204G mutant may be attributed to optimized surface charge distribution, strengthened hydrophobic interactions, and increased tertiary structure stability. Overall, our findings provided valuable insights into enzyme stabilization strategies and supported the industrial production of functional AOS.

## 1. Introduction

Alginate, an important marine polysaccharide, exhibits unique physicochemical properties and bioactivities, showing broad prospects in food, pharmaceuticals, industrial biotechnology, and renewable energy. Alginate is a linear anionic polysaccharide composed of β-D-mannuronic acid (M) and its C5-epimer α-L-guluronic acid (G) linked by 1–4 glycosidic bonds [1]. Alginate oligosaccharides (AOS), degraded products of alginate with a degree of polymerization (DP) of 2–25, display superior solubility, enhanced bioavailability, improved bioactivity, and more consistent product quality [2,3]. Numerous studies have demonstrated that AOS possess various biological activities, including blood glucose-lowering, anti-inflammatory, antibacterial, antioxidant, and antitumor properties [4,5,6,7]. GV-971 (sodium oligomannate) was conditionally approved in China in 2019. Its active ingredient is an alginate oligosaccharide—oligomeric β-1→4 D-mannuronic acid sodium—which ameliorates mild to moderate Alzheimer’s disease by reshaping the gut microbiota and inhibiting neuroinflammation [8]. Furthermore, AOS have also been demonstrated to possess prebiotic activity [9].

Enzymatic catalysis is currently the predominant method for producing functional AOS. Alginate lyases selectively degrade alginate through the β-elimination mechanism, cleaving 1,4-glycosidic bonds and forming a double bond between C4 and C5 at the nonreducing end, yielding unsaturated alginate oligosaccharides (UAOS) [10]. The presence of the unsaturated bonds endows UAOS with distinctive bioactivities, such as lipid-lowering and immunomodulatory effects [11,12]. Moreover, the unsaturated monosaccharides can be converted—either enzymatically or nonenzymatically—into 4-deoxy-L-erythro-5- hexoseulose uronate (DEH). DEH is then reduced by the enzyme DehR to form 2-keto-3-deoxygluconate (KDG), which enters the Entner–Doudoroff (ED) pathway to yield ethanol. In this manner, UAOS serve as a valuable carbon source for bioethanol production and biorefining [13,14].

Alginate lyases have been discovered, cloned, and characterized from a broad array of sources—including brown algae, marine and soil microorganisms, marine invertebrates, Chlorella viruses, and fungi [15]. Based on substrate specificity, alginate lyases are categorized into polyM-specific enzymes (EC 4.2.2.3), polyG-specific enzymes (EC 4.2.2.11), and bifunctional enzymes (EC 4.2.2.-) [16]. According to their mode of action, alginate lyases are classified as either endo-type or exo-type. Endo-type lyases cleave glycosidic bonds randomly in the alginate polymer, resulting in unsaturated oligosaccharides with a DP of 2–6, while exo-type lyases only produce monosaccharides or disaccharides by gradation from the end of the alginate molecule [17]. In the Carbohydrate-Active enZYmes Database, alginate lyases are categorized into polysaccharide lyase families (PL) 5, 6, 7, 8, 14, 15, 17, 18, 31, 32, 34, 36, 39, and 41 [18].

In industrial production, the enzymatic degradation of seaweed biomass is typically conducted at temperatures of 50 °C or higher. Such conditions enhance the solubility of polysaccharides, reduce viscosity, and improve enzyme accessibility to the substrate, thus accelerating the reaction rate [19]. Additionally, high temperatures help inhibit microbial contamination, ensuring greater process stability [20]. Utilizing thermostable alginate lyases allows for continuous high-temperature operations, reduces cooling energy requirements, and ultimately boosts the degradation efficiency. However, most alginate lyases originate from marine environments, exhibiting peak activity at low temperatures and being prone to high-temperature inactivation, with half-lives at 50 °C usually under 10 h due to insufficient thermostability [21,22,23]. For example, the alginate lyase aly-SJ02, isolated from *Pseudoalteromonas* sp. SM0524, exhibited peak activity at 50 °C, with a half-life of 41 min at 40 °C and 20 min at 50 °C [24]. The PL7 family enzyme Aly7B_Wf retained 86.5% and 75.5% of its activity after 24 h at 25 °C and 35 °C, respectively, but rapidly lost stability above 40 °C, retaining only about 40% activity after 6 h at 50 °C [25]. These heat-sensitive properties severely hinder the efficiency of alginate lyases in industrial AOS production, underscoring the urgent need to enhance their thermal stability through protein engineering and other innovative approaches.

In our group’s previous research, the alginate lyase VxAly7B, derived from *Vibrio xiamenensis* QY104, was demonstrated to have excellent alginate degradation ability [26]. VxAly7B contained an N-terminal carbohydrate-binding module (CBM32) and a C-terminal catalytic module (PL7). Experimental data indicated that the presence of CBM32 reduced the enzyme’s thermal stability; when only the catalytic module was retained, the enzyme (VxAly7B-CM) adopted a more compact three-dimensional structure, leading to a marked improvement in heat resistance. In this study, we aim to further enhance the thermal stability of VxAly7B-CM through an effective combinatorial strategy that merges evolutionary coupling analysis and ΔΔG_fold_ evaluation. The combinatorial mutant E188N/S204G demonstrated remarkable improvements in both catalytic activity and thermal stability. Additionally, multiple sequence alignments, structural analysis, and molecular dynamics simulations were performed to elucidate the molecular mechanisms underlying the improved thermal stability of the mutant. This work provided an effective strategy for protein engineering modifications.

## 2. Results

### 2.1. Rational Design of Mutants

**Figure 1:**
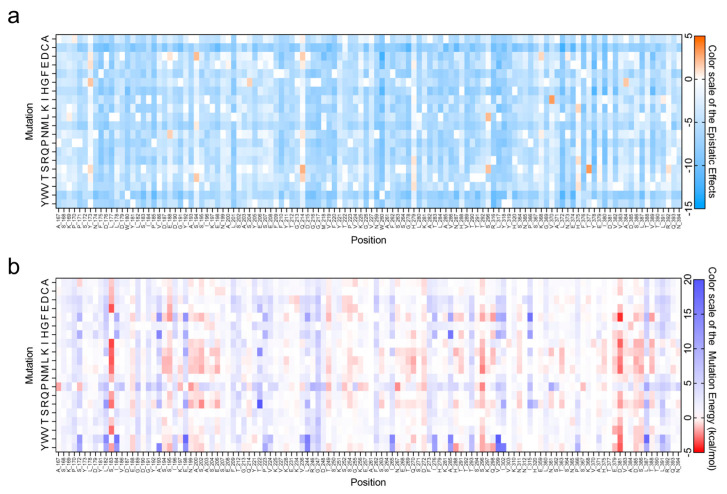
Prediction of mutation effects. (**a**) Epistatic effects of VxAly7B-CM mutants. The data were visualized using GraphPad Prism 8.0, with a color gradient representing values from −15 (blue) to 0 (white) to 5 (orange); (**b**) ΔΔG_fold_ of VxAly7B-CM mutants. The data were visualized using GraphPad Prism 8.0, with a color gradient representing values from −5 (red) to 0 (white) to 20 (purple).

To improve the catalytic performance of the alginate lyase VxAly7B-CM, the protein sequence was first submitted to the EVcouplings web server for evolutionary coupling analysis and assessment of mutation epistatic effects (Figure 1a) [27]. Mutations exhibiting epistatic effect values >0 (indicative of beneficial substitutions) were selected as the basis for rational design. The negative value of ΔΔG_fold_ typically indicates enhanced protein stability [28]. The FireProt web server was utilized to predict the ΔΔG_fold_ values of the mutants (Figure 1b), with a selection threshold of ΔΔG_fold_ < −0.5 kcal/mol [29]. A total of 10 mutants that met the above criteria were prioritized for further investigation, including E188D, E188N, S194M, S204G, Q214S, S296L, K368D, V370I, H375K, and A384G. The mutation epistatic effects and ΔΔG_fold_ values of these mutants are listed in Table S1.

**Figure 2:**
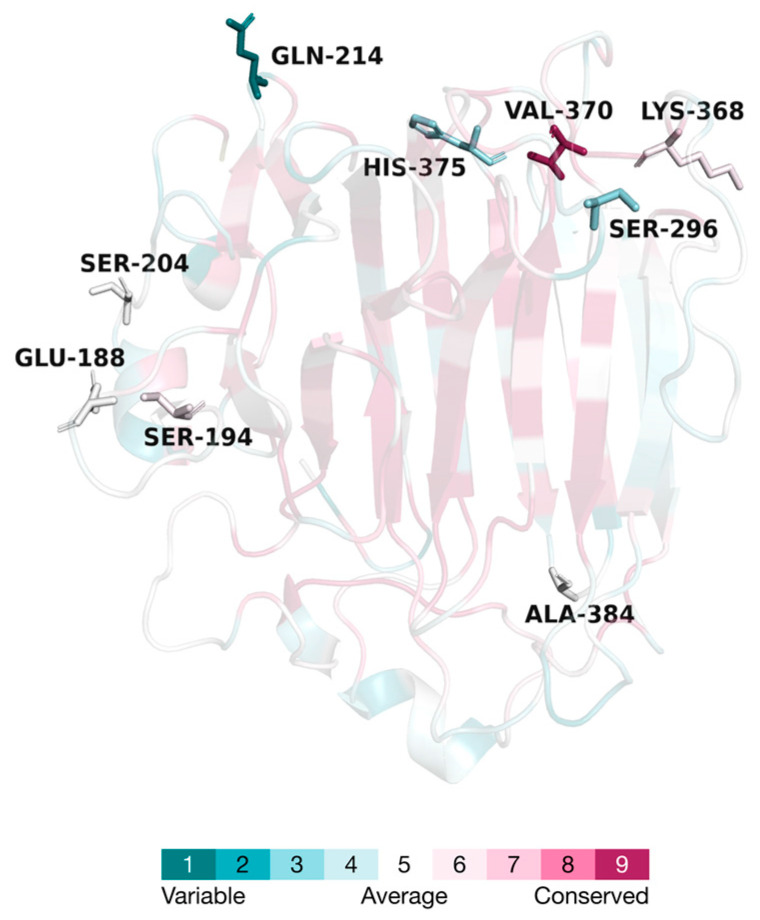
Three-dimensional model and the evolutionary conservation of VxAly7B-CM. The amino acid residues were color-coded according to the conservation grades, with the mutated amino acid residues highlighted in stick models.

The positions of the mutated amino acid residues in the three-dimensional structure of VxAly7B-CM, along with their respective evolutionary conservation grades, are shown in Figure 2. All mutation sites were located on the surface of the protein structure, far from the catalytic active sites and substrate-binding pocket. Except for the V370 site, the other eight sites were in low-conservation regions, indicating that the designed point mutation strategy largely avoided interference with the enzyme’s catalytic function [30]. All mutants were successfully expressed in *E. coli* BL21(DE3) with the correct molecular weight (approximately 33.0 kDa) (Figure S2).

### 2.2. Biochemical Characterization of VxAly7B-CM and Its Mutants

The specific activities and the melting temperature (*T*_m_) of the wild-type VxAly7B-CM (WT) and its mutants are shown in Table 1. Among all mutants, the E188N mutant exhibited a significant enhancement in catalytic activity, reaching 3701.02 U/mg—1.26 times that of the WT. E188D and S204G mutants maintained relative activities of 103% and 95%, respectively. Although the other variants showed various degrees of activity loss, each retained over 65%, with none being completely inactivated. The *T*_m_ values for the E188D, E188N, S204G, S296L, K368D, and V370I increased to various extents compared with the WT. Among these, the S204G mutant exhibited the highest *T*_m_, reaching 50.2 °C, with a Δ*T*_m_ of 3.2 °C. E188N attained a *T*_m_ of 48.9 °C, ranking second only to S204G.

**Figure 3:**
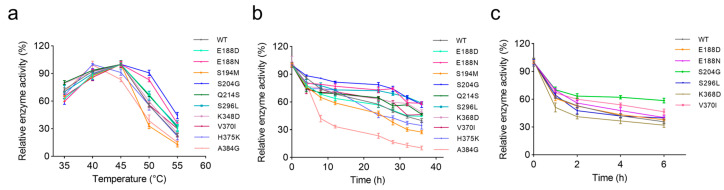
Optimal temperature and thermal stability of VxAly7B-CM and its mutants. (**a**) The optimal temperature of VxAly7B-CM and its mutants; (**b**) the thermal stability of VxAly7B-CM and its mutants at 45 °C; (**c**) the thermal stability of VxAly7B-CM and its mutants at 50 °C.

According to Figure 3a, the WT reached its maximum activity at 45 °C, but its performance dropped rapidly at higher temperatures. Mutants H375K and A384G shifted their optimum to 40 °C, while all other variants maintained a peak at 45 °C, consistent with the WT. Remarkably, both E188N and S204G exhibited over 87% of their maximum activity at 50 °C, showing high-temperature adaptability.

Further assessment of thermal stability was conducted by monitoring enzyme activity retention at 45 °C over incubation periods of 0, 4, 8, 12, 24, 28, 32, and 36 h (Figure 3b). The S204G variant demonstrated the slowest rate of inactivation, maintaining 78.6% and 57.1% activity at 24 and 36 h, respectively. Likewise, E188N and S296L also exhibited a significant improvement in thermal stability within 36 h. In contrast, the A384G variant experienced a rapid decline to 42.0% activity within 8 h, and the stabilities of S194M and H375K also deteriorated significantly after 12 h, with activities remaining below the WT. These results are also highly consistent with the *T*_m_ values.

Based on the above findings, mutants showing significant improvements in thermal stability (E188D, E188N, S204G, S296L, K368D, and V370I) were selected for further characterization. In the accelerated stability test at 50 °C (Figure 3c), the S204G variant exhibited exceptional thermal stability by retaining 60% of its activity after 6 h, considerably outperforming the WT and the other variants, which retained less than 46%. The E188N and V370I mutants also conferred improved stability at 50 °C. Notably, E188N not only exhibited a marked enhancement in catalytic activity but also improved thermal stability at both 45 °C and 50 °C. In a word, while S204G preserved robust stability without substantial activity loss, E188N enhanced both catalytic performance and thermal resilience. Consequently, we constructed the combined mutant E188N/S204G to further optimize modification effects.

### 2.3. Optimal Temperature and Thermal Stability of VxAly7B-CM and the Mutant E188N/S204G

**Figure 4:**
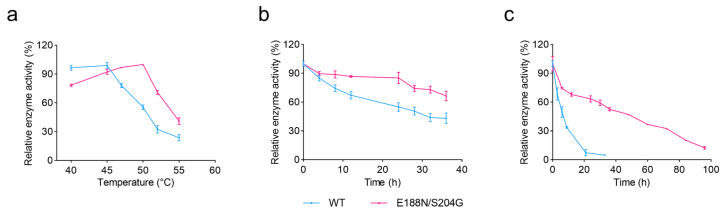
Optimal temperature and thermal stability of VxAly7B-CM and the mutant E188N/S204G. (**a**) The optimal temperature of VxAly7B-CM and the mutant E188N/S204G; (**b**) the thermal stability of VxAly7B-CM and the mutant E188N/S204G at 45 °C; (**c**) the thermal stability of VxAly7B-CM and the mutant E188N/S204G at 50 °C.

As shown in Figure 4a, the optimal reaction temperature for the E188N/S204G mutant was shifted to 50 °C, where its activity reaches 3823.80 U/mg, a 33% increase compared to the WT, and slightly higher than that of the E188N mutant. Moreover, at 52 °C, the E188N/S204G variant maintained 71% of its activity, whereas the WT retains only 32%. The *T*_m_ of the E188N/S204G variant was the highest among all mutants, reaching 52.0 °C—an increase of 5 °C—revealing a marked improvement in structural stability.

Under incubation at 45 °C (Figure 4b), both the WT and the mutant E188N/S204G retained high activity during the initial phase (0–4 h). However, the WT activity declined rapidly, dropping to 67% after 12 h, while E188N/S204G showed a much slower decrease, retaining about 64% activity after 36 h. Under incubation at 50 °C (Figure 4c), the stability disparity between WT and E188N/S204G became even more pronounced: E188N/S204G exhibited a half-life (t_1/2_) of 38.4 h—7.0-fold that of the WT (t_1/2_ = 5.5 h). These results underscore the high-temperature application potential of the E188N/S204G mutant in alginate degradation.

### 2.4. Analysis of Degradation Products

**Figure 5:**
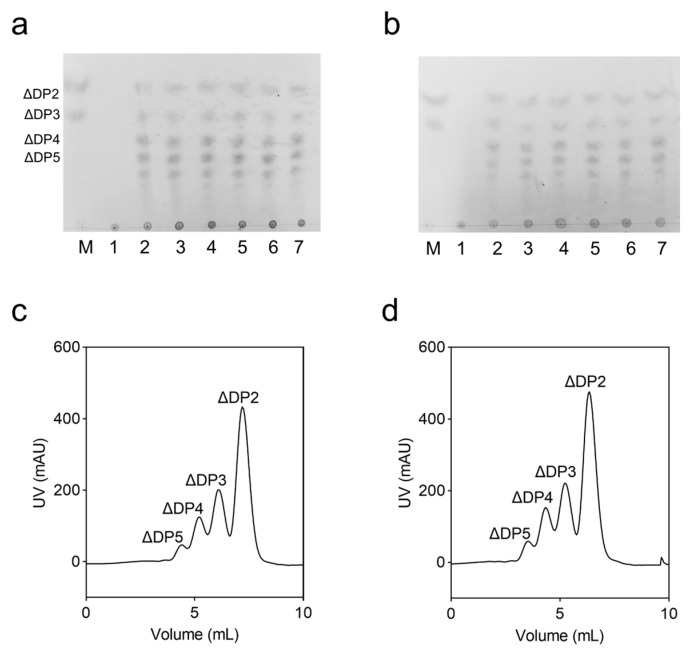
Analysis of degradation products. (**a**) TLC analysis of VxAly7B-CM degradation products (M: Marker, containing unsaturated alginate disaccharides and trisaccharides; lanes 1–7 corresponded to degradation products collected at 0, 1, 5, 15, 30, 60, and 120 min, respectively, ΔDP2, ΔDP3, ΔDP4, and ΔDP5 denoted unsaturated di-, tri-, tetra-, and penta-saccharides, respectively); (**b**) TLC analysis of the E188N/S204G mutant degradation products; (**c**) FPLC analysis of VxAly7B-CM end degradation products; (**d**) FPLC analysis of the E188N/S204G mutant end degradation products.

VxAly7B-CM was an endo-type alginate lyase that generated various sizes of unsaturated oligosaccharides at the early stages of the reaction, with the degradation products primarily consisting of high-molecular-weight sugar chains (Figure 5a). As the reaction progressed, the proportion of smaller oligosaccharides (ΔDP2 and ΔDP3) gradually increased. As shown in Figure 5b, the mutations did not alter the degradation pattern of the enzyme. In addition, final degradation products were analyzed by FPLC (Figure 5c,d) and negative ion ESI-MS (Figure S3). FPLC separation results showed that the final degradation products of WT and the E188N/S204G mutant were ΔDP2, ΔDP3, ΔDP4, and ΔDP5, with the same molar ratios of 2.26:1.96:1.35:1.

### 2.5. Surface Charge and Hydrophobic Distribution of Proteins

**Figure 6:**
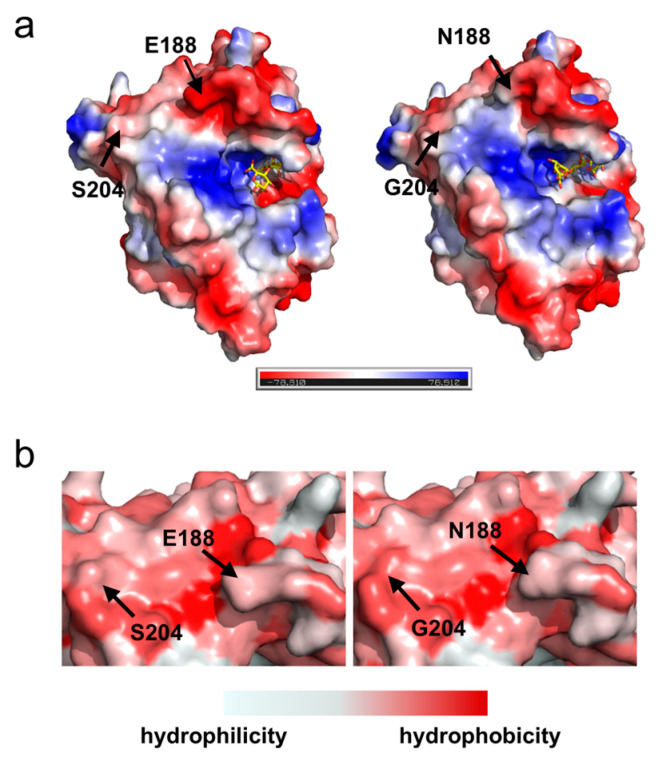
Surface charge and hydrophobicity distribution of proteins: (**a**) Surface charge distribution of VxAly7B-CM and the E188N/S204G mutant (left to right). Yellow sticks represent the tetrasaccharide substrate; (**b**) Surface hydrophobicity distribution of VxAly7B-CM and the E188N/S204G mutant (left to right).

Optimizing the electrostatic charge distribution on the enzyme surface not only enhances its thermal stability but also promotes enzyme–substrate binding, improving catalytic efficiency [31,32,33]. As depicted in Figure 6a, substituting Ser204 with Gly204 did not significantly alter the local charge properties, as both residues are neutral. Interestingly, replacing Glu188 with Asn188 markedly reduced the local negative charge. Structurally, the side chain of Glu residue carries a negatively charged carboxyl group (-COOH), whereas Asn residue features a polar amide group (-CONH_2_); this change diminished the negative charge at position 188. The replacement of Ser204 with Gly204 increased the hydrophobicity of the affected region due to the simpler side chain of the Gly residue, which lacks the polar hydroxyl group present in the Ser residue, thereby promoting the formation of a more compact and stable folded structure (Figure 6b) [34,35].

### 2.6. Analysis of Protein Intramolecular Interaction

**Figure 7:**
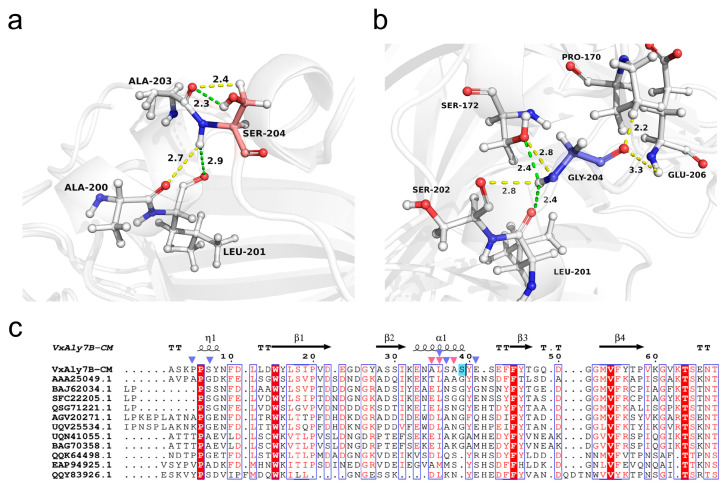
Intramolecular interaction analysis. (**a**) Residue interactions at the 204 site in VxAly7B-CM (relevant amino acid residues are depicted as stick models; green dashed lines represent hydrogen bonds, yellow dashed lines represent polar bonds); (**b**) residue interactions at the 204 site in E188N/S204G; (**c**) sequence alignment of VxAly7B-CM with PL7 family alginate lyases and distribution of secondary structures (position 204 is marked in blue; pink triangles denote amino acids interacting with S204 in WT; purple triangles denote amino acids interacting with G204 in the E188N/S204G mutant).

Analysis of intramolecular interactions in both WT and the mutant E188N/S204G proteins revealed significant alterations upon substituting Ser204 with Gly204, as depicted in Figure 7a,b. In the WT, Ser204 formed hydrogen bonds with neighboring residues Leu201 and Ala203, and also engaged in polar interactions with Ala200 and Ala203. In the E188N/S204G, Gly204 formed hydrogen bonds with Ser172 and Leu201, and the average bond length decreased from 2.6 Å to 2.4 Å. Meanwhile, Gly204 formed new polar bonds with Pro170, Ser172, Ser202, and Glu206. Analysis of the secondary structure of VxAly7B-CM revealed that, compared to Ser204, which interacted only with residues within the α1 helix, Gly204 formed stronger hydrogen bonds and additional polar interactions with residues in both the η1 helix (3_10_ helix) and the α1 helix (Figure 7c). Gly204 likely stabilized the structures of these two flexible helices and enhanced their connection [36,37].

### 2.7. MD Simulations Analysis

**Figure 8:**
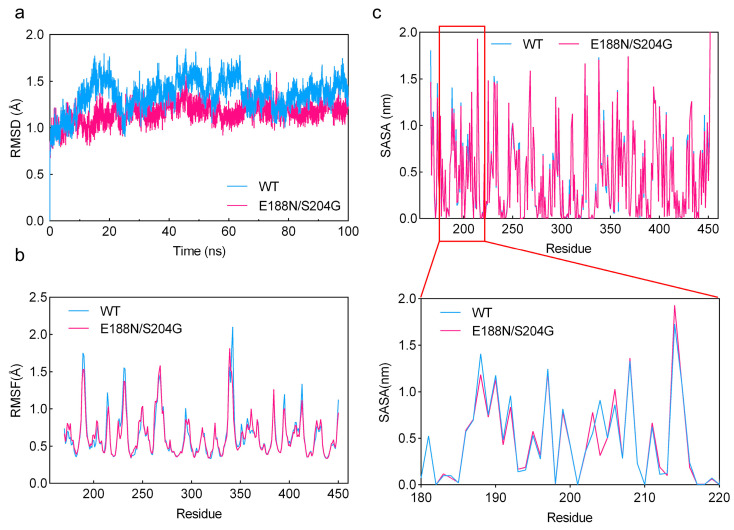
Molecular dynamics simulation of VxAly7B-CM and its mutant E188N/S204G. (**a**) RMSD values of VxAly7B-CM and E188N/S204G during 100 ns MD simulations at 323 K; (**b**) RMSF values of VxAly7B-CM and E188N/S204G during 100 ns MD simulations at 323 K; (**c**) SASA values of VxAly7B-CM and E188N/S204G during 100 ns MD simulations at 323 K.

To systematically evaluate the structural stability, a 100 ns molecular dynamics (MD) simulation was performed under 323 K conditions. As shown in Figure 8a, the backbone RMSD of WT rose from ~0.9 Å at 0 ns to ~1.6 Å, then fluctuated between 1.0 and 1.7 Å from 20 to 100 ns, indicating substantial conformational deviations over the course of the simulation. In contrast, E188N/S204G maintained a narrower RMSD range (~0.9–1.4 Å), with a smoother curve and reduced amplitude of fluctuation, demonstrating enhanced structural integrity and a more compact backbone organization under elevated temperature [38]. The RMSF profiles for both the mutant and WT exhibited similar overall trends, likely due to the inherent stability of VxAly7B-CM, rendering minor stability enhancements less discernible (Figure 8b) [39]. Nevertheless, the RMSF curve of E188N/S204G was consistently below that of WT, especially at peak positions like residues 185–190, 230–235, and 340–350, suggesting smaller residue fluctuations. In conclusion, mutant E188N/S204G had a highly rigid and more stable protein structure.

Solvent-accessible surface area (SASA) measures the area of a molecule’s surface that is accessible to solvent (typically water). During protein folding, hydrophobic residues tend to be buried in the protein core, thereby reducing their SASA and enhancing overall stability [40]. As shown in Figure 8c, the average SASA of the E188N/S204G mutant was lower than that of WT. In particular, around residue 204, the SASA decreased markedly upon mutation, indicating that the side chains in this region relocated into the protein interior. This observation was consistent with the local hydrophobicity increase conferred by the S204G mutation described in Section 2.5—in other words, the S204G mutation drove nearby residues inward, strengthened hydrophobic packing, minimized unfavorable water contacts, and improved protein stability. A similar decrease in SASA was also observed at residue 188.

## 3. Discussion

In this study, we implemented a computational-aided engineering strategy combining evolutionary coupling analysis and ΔΔG_fold_ prediction to simultaneously improve the catalytic activity and thermostability of alginate lyase VxAly7B-CM. Among the engineered variants, E188N demonstrated a 26% activity enhancement alongside superior thermostability, whereas S204G achieved marked thermal stabilization without catalytic trade-offs. The combinatorial mutant E188N/S204G exhibited an enzymatic activity of 3823.80 U/mg and a half-life of 38.4 h at 50 °C, surpassing most reported alginate lyases in both catalytic efficiency and thermal endurance.

Balancing catalytic efficiency and thermal stability remains challenging in enzyme engineering [41,42]. Improving an enzyme’s thermal stability often necessitates a comprehensive understanding of its sequence conservation, three-dimensional structure, and catalytic mechanism to prevent performance loss. For instance, to enhance the thermal stability of the alginate lyase cAlyM through the introduction of disulfide bonds without compromising enzymatic activity, Yang et al. performed analyses of catalytic sites, secondary structures, spatial proximities, and 3D structural flexibility to identify suitable candidate mutations [43]. Based on detailed analyses of the E226K-PM4 binding mode in prereaction-state MD simulation and subsequent three rounds of mutagenesis, Su et al. reengineered the flexible loops and substrate entrance of the catalytic cavity in the PL18 alginate lyase E226K, increasing the half-life at 45 °C from 89 min to 557 min [44].

In protein evolution, residues that interact—especially those close in space or functionally interdependent—often coevolve to preserve structure and activity [45]. Unlike most computational methods that assess each position independently (independent model), EVcouplings can capture dependencies between residues (epistatic model) [46,47]. By combining single-site conservation with pairwise coupling constraints, EVcouplings provides a more comprehensive evaluation of mutation effects. Research had shown that the correlation between the computed ΔE of the epistatic model and experimental measurements of phenotype was more significant than that of the independent model [48].

In this study, EVcouplings was applied to predict evolutionary beneficial mutations, thereby reducing the risk of disrupting key functional interactions. Subsequently, ΔΔG_fold_ calculations were used to screen for mutations that significantly enhance structural stability. Our experimental results indicated that the ten mutations satisfying the criteria of epistatic effects >0 and ΔΔG_fold_ < −0.5 kcal/mol were all located on the protein surface, distant from catalytic active sites and substrate-binding pockets, and exhibited low conservation. Although some variants showed varying degrees of activity loss, each retained over 65% activity, possibly due to evolutionary coupling analysis effectively avoiding perturbations of key functional residues. Notably, E188N exhibited enhanced thermostability (Δ*T*_m_ = 1.9 °C) with a 26% higher specific activity than the wild-type enzyme. S204G preserved robust stability without substantial activity loss. These findings suggested that the integration of the EVcouplings tool with ΔΔG_fold_ calculations can enhance both structural integrity and functional performance of proteins—even in the absence of detailed prior knowledge regarding enzyme architectures or catalytic mechanisms. It provides a straightforward yet effective strategy with broad applicability across diverse protein systems.

Docking simulations with a tetrasaccharide substrate [4-deoxy-L-erythro-hex-4-ene-pyranosyluronate-(mannuronate)(2)-mannuronic acid] revealed that position 188, while not directly involved in substrate binding or located within the catalytic pocket, was situated at the entrance of the catalytic cavity (Figure 6a). In the WT, the pronounced negative charge at the entrance may impede the entry of similarly negatively charged substrates due to electrostatic repulsion. While the E188N mutation diminished the negative charge at the entrance, potentially facilitating substrate access and enhancing catalytic activity [49,50].

In the E188N/S204G mutant, substituting Ser204 with Gly204 eliminated the polar hydroxyl group from the side chain, increasing local hydrophobicity and promoting the inward movement of adjacent residues to enhance hydrophobic core packing—factors crucial for improving protein thermostability. Additionally, Gly204 formed stronger interactions with neighboring residues and acted as a linker between the η1 and α1 helical structures. This connectivity likely contributed to stabilizing flexible helices, reducing their susceptibility to distortion under elevated temperatures. Furthermore, the absence of a side chain in Gly reduced steric hindrance, increasing regional flexibility. From a dynamic perspective, those changes allowed the η1 and α1 helices to coordinate more effectively during protein folding or functional motion, minimizing conformational conflicts and enhancing overall stability [51,52]. Therefore, we speculated that the multi-optimization of local charge distribution, intramolecular interaction, and three-dimensional structure conformation enabled the E188N/S204G mutant to maintain high catalytic efficiency over extended periods at elevated temperatures.

In conclusion, the superior enzymatic performance and thermal stability of the E188N/S204G mutant established a solid foundation for its application in AOS production. Furthermore, the rational design strategy employed in this study provided valuable insights and a versatile methodology for engineering other thermosensitive enzymes, broadening the scope of protein engineering for industrial biotechnology.

## 4. Materials and Methods

### 4.1. Bacterial Strains and Chemicals

The recombinant plasmid pET24a(+)-VxAly7B-CM utilized in this study was maintained in our laboratory and transformed into *E. coli* JM109 for amplification. The domain organization and amino acid numbering of VxAly7B-CM are shown in Figure S1. For the expression of VxAly7B-CM and its mutants, an expression system was established using *E. coli* BL21 (DE3) as the host strain. The molecular biology reagents used in this study were obtained from commercial sources, including the TIANGEN Biotech plasmid extraction kit (Beijing, China), the ToYoBo KOD One™ PCR Master Mix (Beijing, China), the OMEGA Bio-tek DNA gel extraction kit (Guangzhou, China), and the Novoprotein homologous recombination kit (Suzhou, China). Sodium alginate was purchased from Sigma-Aldrich (St. Louis, MO, USA), and all other chemicals were of analytical grade and commercially available.

### 4.2. Mutation Site Selection and Construction

#### 4.2.1. Rational Design of Candidate Mutants

The VxAly7B-CM protein sequence was submitted to the AlphaFold 3 online server (https://deepmind.google/technologies/alphafold/alphafold-server/, accessed on 25 September 2024) for three-dimensional structural prediction. The model quality was further assessed using the SAVES 6.0 (Verify3D tool) online server (https://saves.mbi.ucla.edu/, accessed on 25 September 2024) [53]. The EVcouplings web server was used to evaluate the effect of mutations. Based on multiple sequence alignments (MSAs) of numerous homologous sequences, EVcouplings constructs the Potts model with regularized maximum pseudolikelihood to identify single-site constraints and pairwise coupling constraints, calculating the mutation epistatic effects [48]. Using the FireProt 2.0 web server (https://loschmidt.chemi.muni.cz/fireprotweb/,accessed on 9 October 2024), the three-dimensional structure of VxAly7B-CM was analyzed with FoldX to predict ΔΔG_fold_ values based on the calculation of the empirical force field [54]. GraphPad Prism 8.0 was utilized to visualize the anticipated effects of these mutations. The Consurf server (https://consurf.tau.ac.il/, accessed on 21 October 2024) was employed to analyze the conservation of amino acid residues based on multiple sequence alignment.

#### 4.2.2. Construction of Mutants by Site-Directed Mutagenesis

Site-directed mutagenesis was conducted using the plasmid pET24a(+)-VxAly7B-CM as the template, with mutation-specific primers detailed in Table S2. The amplified PCR products were digested with the restriction enzyme *Dpn* I to eliminate the methylated template plasmid, followed by Gibson recombination and transformed into *E. coli* JM109. Single colonies were selected and cultured overnight in Luria–Bertani (LB) solid medium containing 30 μg/mL kanamycin sulfate. The plasmids were extracted for DNA sequencing. Primer synthesis and DNA sequencing services were provided by Tsingke Biotechnology Co., Ltd. (Tianjin, China).

#### 4.2.3. Expression and Purification of VxAly7B-CM and Its Mutants

The recombinant plasmid was transformed into *E. coli* BL21(DE3) for protein expression. A single colony was inoculated into 5 mL of LB liquid medium containing 30 μg/mL kanamycin sulfate and incubated at 37 °C for 8 h. This culture was then diluted 1:100 into fresh LB medium with the same antibiotic concentration and incubated at 37 °C with shaking at 220 rpm until the OD_600_ reached 0.4–0.6. Protein expression was induced by adding isopropyl β-D-1-thiogalactopyranoside (IPTG) to a final concentration of 100 μM, followed by incubation at 18 °C with shaking at 160 rpm for 24 h.

The recombinant VxAly7B-CM and its mutants, each containing a C-terminal (His)_6_ tag, were purified using a HisTrap HP chromatography column (GE Healthcare Life Sciences, Piscataway, USA). The protein storage buffer consisted of 20 mM phosphate buffer (PB) at pH 7.6, supplemented with 500 mM NaCl. To remove unbound proteins, the column was washed with buffer containing 25 mM imidazole, and the target proteins were eluted with buffer containing 100 mM imidazole. The purity and molecular weight of the proteins were assessed by sodium dodecyl sulfate-polyacrylamide gel electrophoresis (SDS-PAGE) on a 12% (*w*/*v*) resolving gel. Protein concentrations were determined using the BCA Protein Assay Kit (Beyotime Biotechnology Co., Ltd., Shanghai, China) [55].

### 4.3. Biochemical Characterization of VxAly7B-CM and Its Mutants

#### 4.3.1. Enzyme Activity Assay

Enzyme activity was assessed using the A_235_ method, which monitors the increase in absorbance at 235 nm resulting from the formation of unsaturated double bonds in the degradation products [56]. The reaction mixture (1 mL) consisted of 100 μL of appropriately diluted enzyme solution and 900 μL of pre-warmed substrate solution [0.3% (*w*/*v*) alginate substrate, 20 mM PB, 500 mM NaCl, pH 7.6]. The reaction was conducted at 45 °C for 10 min, and absorbance changes at 235 nm were recorded. One unit (U) of enzyme activity was defined as the amount of enzyme required to increase the absorbance at 235 nm by 0.1 per min.

#### 4.3.2. Optimal Temperature and Thermal Stability Assay

To determine the optimum temperature, the activities of alginate lyase were measured at various temperatures ranging from 35 to 55 °C. The highest observed activity was defined as 100%, and relative activities at each temperature were calculated accordingly.

The melt temperature (*T*_m_) values were determined using the micro-differential scanning fluorometer Prometheus NT.48 (NanoTemper Technologies GmbH, Munich, Germany), which can monitor changes in the intrinsic fluorescence of tryptophan and tyrosine residues during protein unfolding. Protein samples were diluted to a concentration of 1 mg/mL and centrifuged at 4 °C at 12,000 rpm for 10 min to remove impurities. The scanning temperature was gradually increased from 25 °C to 90 °C at a rate of 1 °C/min. Each sample was measured in triplicate.

To determine the thermal inactivation half-life (t_1_/_2_), the activity of the untreated alginate lyases served as the standard and was designated as 100%. The activities at different times were then expressed as relative (%) values compared with the untreated control. The data obtained were analyzed using GraphPad Prism 8.0 software to calculate t_1_/_2_.

### 4.4. Analysis of Degradation Product

The changes in degradation products over time were analyzed by thin-layer chromatography (TLC). Enzymatic hydrolysis of a 0.3% (*w*/*v*) alginate substrate was conducted at 45 °C. Samples were collected at 0, 1, 5, 15, 30, 60, and 120 min, then boiled for 10 min to terminate the reaction. In this procedure, the developing solvent consisted of n-butanol, formic acid, and water in a volumetric ratio of 4:6:1. The staining reagent was an aniline-diphenylamine solution composed of 4 g diphenylamine, 4 mL aniline, 20 mL 85% (*v*/*v*) phosphoric acid, and 200 mL acetone.

The final degradation products were analyzed by fast protein liquid chromatography (FPLC) and negative-ion electrospray ionization mass spectrometry (ESI-MS). A 0.3% (*w*/*v*) alginate substrate solution was supplemented with enzymes to a final concentration of 20 U/mL and incubated at 45 °C for 12 h to ensure complete substrate degradation, and then terminated by boiling for 10 min. Separation was performed using a Superdex peptide 10/300 GL column (GE Healthcare, USA) with 0.2 M NH_4_HCO_3_ as the mobile phase at a flow rate of 0.2 mL/min, monitoring absorbance changes at 235 nm by FPLC. Fractions corresponding to the chromatographic peaks were collected, mixed in a 1:1 volume ratio with acetonitrile, and analyzed by negative-ion ESI-MS over an m/z range of 0–1000. Ranges without significant sample peaks are not shown.

### 4.5. Structural Analysis and Molecular Dynamics Simulations

#### 4.5.1. Protein Structure Visualization and Analysis

PyMOL 2.5.0 software was used to visualize the structure of VxAly7B-CM and its mutant, and to analyze the surface charge and hydrophilic distribution of the proteins. DynaMut (https://biosig.lab.uq.edu.au/dynamut2/, accessed on 14 January 2025) and Discovery Studio Visualizer (https://discover.3ds.com/discovery-studio-visualizer-download, accessed on 22 April 2025) were utilized to assess inter-residue interactions within the protein structure [57,58]. The amino acid sequence of VxAly7B-CM, along with those of other PL7 family alginate lyases, was uploaded to Clustal Omega for multiple sequence alignment [59]. The alignment results were further visualized using ESPript 3.0 (https://espript.ibcp.fr/ESPript/cgi-bin/ESPript.cgi, accessed on 12 February 2025), and secondary structure analysis was performed using VxAly7B-CM as the template [60].

#### 4.5.2. Molecular Docking

The mannan tetramer (PDB: 4F13) was docked into VxAly7B-CM using AutoDock Vina [61,62,63]. The docking box was set at the predicted conserved catalytic pocket, and the top 10 binding conformations with the lowest binding free energy were selected for analysis.

#### 4.5.3. Molecular Dynamics Simulations

To further assess the structural flexibility of the alginate lyase, molecular dynamics (MD) simulations were performed using GROMACS with the Amber ff99SB force field [64,65]. The system was solvated using the SPC/E water model. To maintain overall charge neutrality, Na^+^ and Cl^−^ ions were incorporated based on the Coulomb potential grid. The entire system was energy-minimized using a combined steepest descent method to eliminate unfavorable atomic contacts. The system was annealed at 318 K for 1 ns with weak restraints applied to the protein. Once a uniform density was achieved during the heating phase, the system was equilibrated at a target pressure of 1.0 bar. MD simulations were then performed at 323 K and 1.0 bar for 100 ns, and the root-mean-square fluctuations (RMSF), root-mean-square deviations (RMSD), and solvent-accessible surface area (SASA) were calculated.

## Figures and Tables

**Table 1:** Characteristics of VxAly7B-CM and its mutants.

Name	*T*_m_ (°C)	Specific Activity (U/mg)
WT	47.0	2935.76 ± 37.40
E188D	48.1	3096.17 ± 77.98
E188N	48.9	3701.02 ± 118.17
S194M	46.4	2799.74 ± 57.95
S204G	50.2	2812.01 ± 76.20
Q214S	46.6	1975.93 ± 55.40
S296L	48.5	2492.81 ± 141.51
K368D	47.8	1945.44 ± 32.79
V370I	48.4	2163.29 ± 96.81
H375K	47.2	2429.83 ± 80.60
A384G	43.7	2661.22 ± 90.39
E188N/S204G	52.0	3832.80 ± 17.84 ^#^

Notes: Specific activity marked with # was measured at 50 °C, while unmarked specific activities were measured at 45 °C.

## Data Availability

All data obtained during this study are available from the corresponding author on reasonable request.

## Supplementary Materials

The following supporting information can be downloaded at https://www.mdpi.com/article/10.3390/md23050198/s1; Table S1: Candidate mutants data predicted using the EVcouplings and FireProt; Table S2: PCR primers for the mutants of VxAly7B-CM; Figure S1: Domain structure of full long VxAly7B and the catalytic domain VxAly7B-CM; Figure S2: Purification of VxAly7B-CM and its mutants; Figure S3: End products of VxAly7B-CM and its mutant E188N/S204G analyzed by negative ion ESI-MS.

## Abbreviations

The following abbreviations are used in this manuscript:

AOS	Alginate oligosaccharides
*T*_m_	Melting temperature
DP	Degree of polymerization
UAOS	Unsaturated alginate oligosaccharides
DEH	4-deoxy-L-erythro-5- hexoseulose uronate
ED	Entner–Doudoroff
PL	Polysaccharide lyase families
MD	Molecular dynamics
RMSD	Root-mean-square deviations
RMSF	Root-mean-square fluctuations
SASA	Solvent-accessible surface area
IPTG	Isopropyl β-D-1-thiogalactopyranoside
TLC	Thin-layer chromatography
FPLC	Fast protein liquid chromatography
ESI-MS	Electrospray ionization mass spectrometry
SDS-PAGE	Sodium dodecyl sulfate-polyacrylamide gel electrophoresis
