# Supplementary material for: Improvement of Catalytic Activity and Thermostability of Alginate Lyase VxAly7B-CM via Rational Computational Design Strategies
Source: Mar Drugs. 2025 May 1;23(5):198. doi: 10.3390/md23050198 (PMC12112969; doi:10.3390/md23050198)
Supplement: Supplementary file 1 [file marinedrugs-23-00198-s001.zip › marinedrugs-3579273-supplementary.pdf]

Supplementary Materials

Improvement of Catalytic Activity and Thermostability of Alginate Lyase VxAly7B-CM via Rational Computational Design Strategies

Xin Ma [1,2,3,4], Ke Zhu [1,2,3,4], Kaiyang Wang [1,2,3,4], Wenhui Liao [1,2,3,4], Xiaohan Yan [5,6], Wengong Yu [1,2,3,4], Weishan Wang [1,5,6,7,*], and Feng Han [1,2,3,4,*]

1 School of Medicine and Pharmacy, Ocean University of China, Qingdao 266003, China; xma1221@163.com (X.M.);
2 Laboratory for Marine Drugs and Bioproducts, Qingdao Marine Science and Technology center, Qingdao 266237, China
3 Key Laboratory of Marine Drugs, Ministry of Education, Qingdao 266003, China
4 Shandong Key Laboratory of Glycoscience and Glycotherapeutics, Qingdao 266003, China
5 State Key Laboratory of Microbial Diversity and Innovative Utilization, Institute of Microbiology, Chinese Academy of Sciences, Beijing, 100101, China
6 University of Chinese Academy of Sciences, Beijing, 100049, China
7 Beijing Key Laboratory of Genetic Element Biosourcing & Intelligent Design for Biomanufacturing, Beijing 100101, China
*Correspondence: fhan@ouc.edu.cn (F.H.); wangws@im.ac.cn (W.W.); Tel.: +86-532-82032067 (F.H.)

Table S1. Candidate mutants data predicted using the EVcouplings and FireProt

**Name**	**Mutation epistatic effects**	**$\Delta\Delta G_{fold}$**
WT	0	0
E188D	0.797	- 0.53
E188N	1.487	- 0.64
S194M	0.694	- 1.43
S204G	1.423	- 0.69
Q214S	2.471	- 0.55
S296L	1.991	- 1.93
K368D	1.251	- 0.58
V370I	3.160	- 0.73
H375K	1.332	- 0.70
A384G	1.791	- 1.15

Table S2. PCR primers for the mutants of VxAly7B-CM

**Primer name**	**Primer sequences (5′-3')**
E188D-F	CAGTCGACGATGGTGATGGGTATGCCAGCTC
E188D-R	CATCACCATCGTCGACTGGAATACTTAGGTACCAG
E188N-F	AGTCGACAATGGTGATGGGTATGCCAGCTC
E188N-R	CCATCACCATTGTCGACTGGAATACTTAGGTACCAG
S194M-F	GTATGCCATGTCGATCAAAGAAAATGCTTTATCTGCG
S194M-R	TTGATCGACATGGCATACCCATCACCCTCG
S204G-F	TATCTGCGGGTTATGAAAGCGAATTTTTTATACTGGCCAG
S204G-R	GCTTTCATAACCCGCAGATAAAGCATTTTCTTTGATCG
Q214S-F	ATACTGGCTCTGATGGGGGAATGGTTTTTATACACCAG
Q214S-R	TTCCCCCATCAGAGCCAGTATAAAAAATTCGCTTTCATAACT
S296L-F	GAGTGATCTGAAACAAGTTGGAAGAATCGTGATCG
S296L-R	CAACTTGTTTCAGATCACTCGTCGTTGTTGTGAC
K368D-F	TCCGTCAGATGGGGTGGCATTAAATGAGCAT
K368D-R	GCCACCCCATCTGACGGATTAGACGTACTATTAAGCT
V370I-F	CAAAAGGGATTGCATTAAATGAGCATTTCACGTATGAAATT
V370I-R	TTAATGCAATCCCTTTTGACGGATTAGACGTAC
H375K-F	TAAATGAGAAATTCACGTATGAAATTGATGTAGTTGCTG
H375K-R	TACGTGAATTTCTCATTTAATGCCACCCCTTTTG
A384G-F	TGTAGTTGGTGATTCACTTACCGTAACTCTTCGC
A384G-R	AGTGAATCACCAACTACATCAATTTCATACGTGAAATGCT

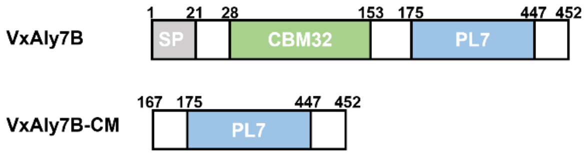

Figure S1. Domain structure of full length VxAly7B and the catalytic domain VxAly7B-CM.

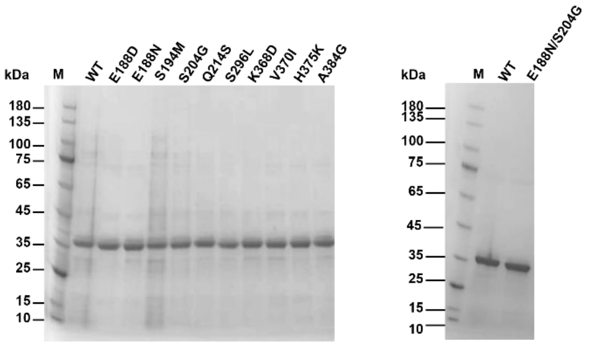

Figure S2. Purification of VxAly7B-CM and its mutants.

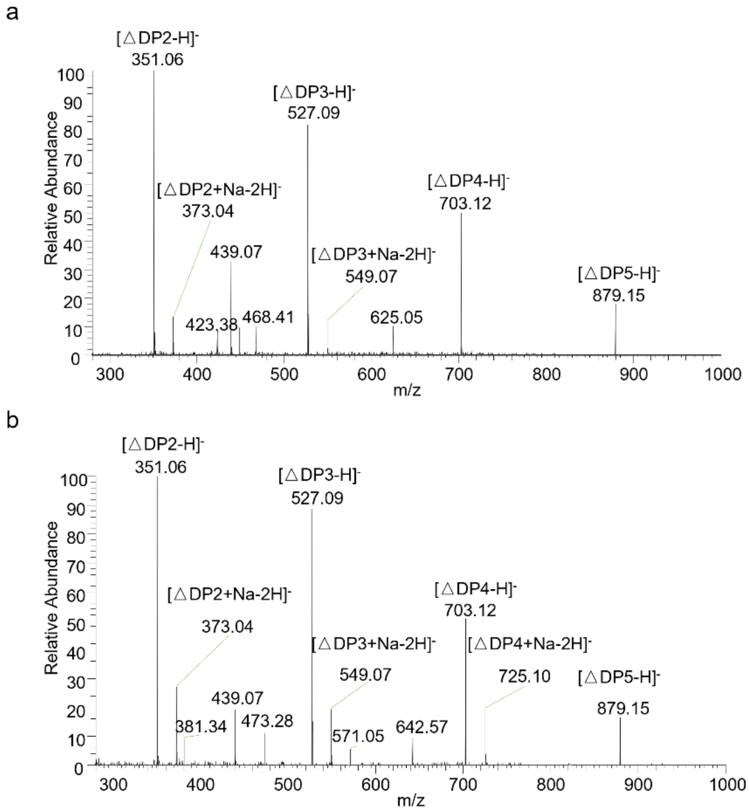

Figure S3. End products of VxAly7B-CM and its mutant E188N/S204G analyzed by negative ion ESI-MS: (**a**) End products of VxAly7B-CM; (**b**) End products of the mutant E188N/S204G.
